# Single-cell atlas reveals cellular heterogeneity and *BMP5*-mediated regulation of adipogenic differentiation in sheep adipose tissue

**DOI:** 10.1038/s42003-026-09581-3

**Published:** 2026-01-21

**Authors:** Jiangbo Cheng, Kunchao Han, Dan Xu, Huibin Tian, Xiaoxue Zhang, Liming Zhao, Xiaobin Yang, Deyin Zhang, Kai Huang, Yukun Zhang, Yuan Zhao, Xiaolong Li, Quanzhong Xu, Zongwu Ma, Weiwei Wu, Jianlin Wang, Fadi Li, Weimin Wang

**Affiliations:** 1 State Key Laboratory of Herbage Improvement and Grassland Agro-ecosystems; Key Laboratory of Grassland Livestock Industry Innovation, Ministry of Agriculture and Rural Afairs; Engineering Research Center of Grassland Industry, Ministry of Education; College of Pastoral Agriculture Science and Technology, Lanzhou University, Lanzhou, China; 2 College of Animal Science and Technology, Gansu Agricultural University, Lanzhou, China; 3 Xinjiang Academy of Animal Sciences, Urumqi, Xinjiang China

**Keywords:** Transcriptomics, Animal breeding

## Abstract

Adipose tissue is a heterogeneous multifunctional organ, and understanding depot-specific cellular and molecular diversity reveals functional differences. Here, We construct a single-cell atlas of major adipose depots in sheep, providing foundational data for understanding regional fat deposition. We identify depot-specific adipocytes, with visceral fat (*ACSS3*+ADC3) and subcutaneous fat (*SCD*+ADC2) showing distinct adipocyte populations, and tail fat containing a specific type of fibroadipogenic progenitor cells (*PDGFRA*+ASPC2). Focusing on the unique tail fat of fat-tailed sheep, we conduct longitudinal developmental analyses and identify the BMP signaling pathway as a key upstream regulator of adipogenesis, with bone morphogenetic protein 5 (*BMP5*) as a critical ligand. We show that knockdown of *BMP5* significantly reduces triglyceride accumulation in adipocytes. Collectively, this study indicates that subcutaneous fat is primarily involved in lipid metabolism, whereas visceral fat is linked to metabolic and immune homeostasis. Moreover, *BMP5* was identified as a key candidate gene regulating tail fat development. These findings provide potential molecular targets for regulating fat deposition in livestock breeding and offer a valuable resource for studying adipose tissue biology in large mammals.

## Abstract (web summary)

Single-cell atlas reveals depot-specific cellular diversity of sheep adipose tissues, identifying BMP5 as a key candidate ligand gene regulating tail fat development.

## Introduction

To withstand cold, food shortages, and other adverse environments, animals have evolved related functions or organs for energy storage^1^. Adipose tissue is an important site for energy storage and plays a significant role in whole-body energy homeostasis and metabolic regulation^2^. Dysfunction of adipose tissue often leads to metabolic disorders, accompanied by conditions such as obesity, insulin resistance, and type 2 diabetes^3^. During development, adipose tissue is highly plastic and expands or contracts based on energy supply and demand. During its dynamic changes, its function is influenced by the types of resident cells^4^.

The diversity of adipose tissue has been a topic of interest for researchers for a long time and is generally defined by its anatomical location and origin. Adipose tissue is generally classified into white, beige, and brown adipose tissue. Both brown and beige adipocytes function in thermogenesis, characterized by abundant mitochondria and high uncoupling protein 1 (*UCP1*) expression^5^. Unlike brown adipocytes, beige adipocytes can arise from white adipocytes in response to cold exposure or norepinephrine stimulation and revert to white adipocytes once the stimulus is removed^6,7^. White fat is the primary energy storage tissue that is widely distributed throughout the body^8^. White fat is further classified into visceral fat and subcutaneous fat based on its local deposition. Subcutaneous fat has a stronger ability to deposit fat and is keen on absorbing free fatty acids and triglycerides, while visceral fat has a higher metabolic activity and tends to transmit pro-inflammatory signals^9,10^. Compared to humans and mice, animals surviving in complex environments possess more specialized fat reserves, such as the humps of camels and the tail fat of sheep (*Ovis aries*)^11^.

Sheep are one of the major sources of animal products and an important biomedical model for investigating human diseases and biological mechanisms. They have been extensively utilized in studies of immune regulation^12^, developmental biology^13^, and adipose metabolism^14^. Among these biological systems, adipose tissue plays a central role in both energy homeostasis and metabolic adaptation. The adipose tissue of fat-tailed sheep often exhibits powerful energy storage and plasticity, with each fat depot displaying distinct gene expression under different external conditions^15^. As humans currently face the threats of obesity and related metabolic syndromes, the demand for low-fat livestock products is increasing. Tail fat, as a unique fat depot in fat-tailed sheep, has become a research focus for reducing its local deposition^16,17^. Our previous studies demonstrated that fat deposition in sheep is negatively associated with feed efficiency, suggesting that limiting fat accumulation could be an effective strategy to improve agricultural productivity and production efficiency^18^. Previous study results indicated that, based on whole-genome selective sweep analyses of fat-tailed and thin-tailed sheep, bone morphogenetic protein 2 (*BMP2*) and platelet-derived growth factor D (*PDGFD*) were identified as candidate genes involved in preadipocyte differentiation^15^. Despite anatomical differences between sheep and humans, the relevant regulatory genes (*BMP2*/*PDGFD*) are conserved in human adipose regulation, suggesting that sheep represent a suitable model for studying fat deposition^19,20^. Studies on ovine visceral fat have shown that, during development, genes associated with inflammatory responses are upregulated, along with increased expression of antigen-processing-related genes such as CD74 molecule (*CD74*) and IFI30 lysosomal thiol reductase (*IFI30*)^21^. These studies highlight the importance of investigating sheep fat depots for understanding sheep diseases and the regulation of fat deposition^15,22,23^. Furthermore, the uniqueness of sheep tail fat, which is absent in both humans and traditional laboratory animals, endows it with irreplaceable research value, and investigating this depot can provide novel insights into the evolutionary adaptation of adipose tissue and the tissue-specific regulation of fat deposition^24^. Currently, research on sheep adipose tissues remains at the bulk RNA-seq level, where RNA from all cells is mixed, masking cellular heterogeneity. Model animals have complete annotation of fat depots at the single-cell level^25–27^, but sheep lack this, which will hinder our understanding of the molecular mechanism of local deposition of fat depots in sheep.

Here, we report the cellular atlas of white fat depots from four anatomical locations in Hu sheep using single-cell RNA-seq. Hu sheep, a fat-tailed sheep breed unique to China, are characterized by high fertility and stress resistance, widely distributed in various environments^28,29^. Our dataset characterizes the cellular atlas and interaction pathways of subcutaneous and visceral fat in sheep. Additionally, we explored the cellular heterogeneity and functions during tail fat development across 4 developmental stages in sheep. Importantly, cell experiments and RNA-seq were used to confirm the regulatory role of the BMP pathway (*BMP5*) in tail fat cell differentiation and development. In summary, this study expands the annotation of sheep fat depots at single-cell resolution, identifies the cellular interaction pathways influencing tail fat deposition, and provides a data foundation for future research on sheep fat depots.

## Results

### Spatiotemporal single-cell atlas of sheep adipose tissue

**Fig. 1:**
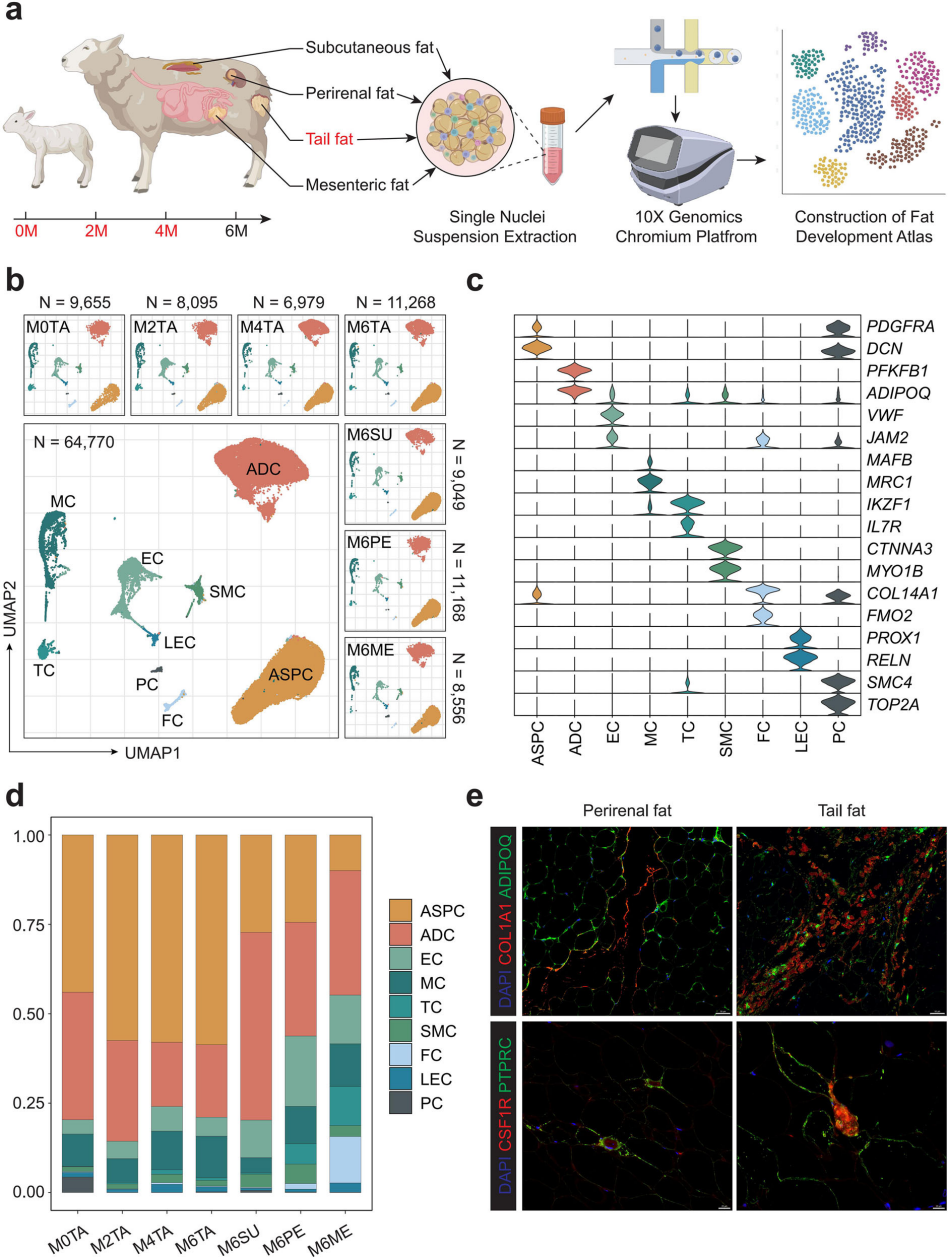
SnRNA-seq showed the main cell types in different fat depots of sheep. **a** Schematic diagram of the experimental pipeline. Tail fat (M6TA), dorsal subcutaneous fat (M6SU), perirenal fat (M6PE), and mesenteric fat (M6ME) were isolated from 6-month Hu sheep, while tail fat was also isolated from 0 (M0TA), 2 (M2TA), and 4 (M4TA) months Hu sheep. Following nuclei isolation, samples were subjected to 10X Genomics library construction and sequencing, and subsequent analyses were performed as described in the Materials and Methods section. **b** The Uniform Manifold Approximation and Projection (UMAP) of cell types in sheep fat, along with the UMAP and cell numbers of the seven groups. ASPC adipose stem and progenitor cells, ADC adipose cells, EC endothelial cells, MC macrophage cells, TC T cells, SMC smooth muscle cells, LEC lymphatic endothelial cells, PC proliferating cells, FC fibroblast cells. **c** Violin plots show genes that are specifically highly expressed in different cell types. **d** The bar graph shows the cell type composition and proportion in all groups revealed by snRNA-seq. **e** Identification of major cell populations based on immunofluorescence staining. *COL1A1* represents ASPC, *ADIPOQ* represents ADC, *CSF1R* represents MC, and *PTPRC* represents TC. Scale bar represents 50 μm (up) and 20 μm (down).

To construct a map of cell populations in different regions and developmental stages of adipose tissue in sheep, we isolated dorsal subcutaneous fat (subcutaneous fat), tail fat (subcutaneous fat), mesenteric fat (visceral fat), and perirenal fat (visceral fat) from 6-month-old sheep. Additionally, we collected tail fat at 0, 2, and 4 months (Fig. 1a). A total of 64,770 cells passed quality control for subsequent analysis, including 35,997 cells from tail fat, 9049 cells from dorsal subcutaneous fat, 11,168 cells from perirenal fat, and 8556 cells from mesenteric fat (Fig. 1b). Unsupervised clustering followed by Uniform Manifold Approximation and Projection (UMAP) dimensionality reduction identified nine clusters (Supplementary Fig. S1a). Based on the expression patterns of known cell type marker genes and Gene Ontology (GO) functional enrichment entries (Fig. 1c, Supplementary Fig. S1b and Supplementary Data 1), the clusters were annotated, primarily including adipose stem and progenitor cells (ASPC), adipocyte cells (ADC), macrophage cells (MC), endothelial cells (EC), smooth muscle cells (SMC), T cells (TC), fibroblast cells (FC), lymphatic endothelial cells (LEC), and proliferating cells (PC) (Fig. 1b). Marker genes for the same cell type exhibited similar expression patterns across different tissues and developmental stages (Supplementary Fig. S1c and Supplementary Data 2). For example, cluster 1 was annotated as ASPC with high expression of platelet-derived growth factor receptor alpha (*PDGFRA*) and decorin (*DCN)*, which are primarily involved in cell growth and differentiation^25,30^. Cluster 2 was defined as ADC highly expressing adiponectin (*ADIPOQ*) and lipase E, hormone-sensitive type (*LIPE*), primarily associated with lipid metabolism functions^25,31^. Clusters 4 and 5 were identified as immune cells, characterized by high expression of interleukin 7 receptor (*IL7R*), protein tyrosine phosphatase receptor type C (*PTPRC*), and colony-stimulating factor 1 receptor (*CSF1R*). These cells are involved in regulating immune responses as well as the proliferation and differentiation of immune cells^32^.

We further compared cell type proportions across different adipose tissues and developmental stages (Fig. 1d and Supplementary Data 3). A higher proportion of ASPC was observed in the tail adipose depot, suggesting a greater adipogenic potential. TC were more abundant in perirenal and mesenteric adipose tissues, which is consistent with the typical pattern of visceral fat. ADC constituted the highest proportion in the dorsal subcutaneous fat tissue, indicating a strong capacity for local lipid deposition and potentially contributing to maintaining body temperature. Notably, a higher proportion of proliferating cells expressing DNA topoisomerase II alpha (*TOP2A*) was found in 0-day-old adipose tissue, reflecting enhanced proliferative cell activity at this stage. Immunofluorescence analysis corroborated the snRNA-seq (single-nucleus RNA-seq) data, revealing a higher abundance of ASPC in the tail adipose depot, as evidenced by stronger collagen type I alpha 1 chain (*COL1A1*) expression (Fig. 1e). Conversely, *PTPRC* expression, a marker gene for TC, was elevated in perirenal fat. The marker gene *ADIPOQ* for ADC and the marker gene *CSF1R* for MC were expressed in both tissues. The immunofluorescence results were consistent with the snRNA-seq data analysis, further confirming the reliability and accuracy of our data.

### Cellular heterogeneity of sheep ASPC and ADC

**Fig. 2:**
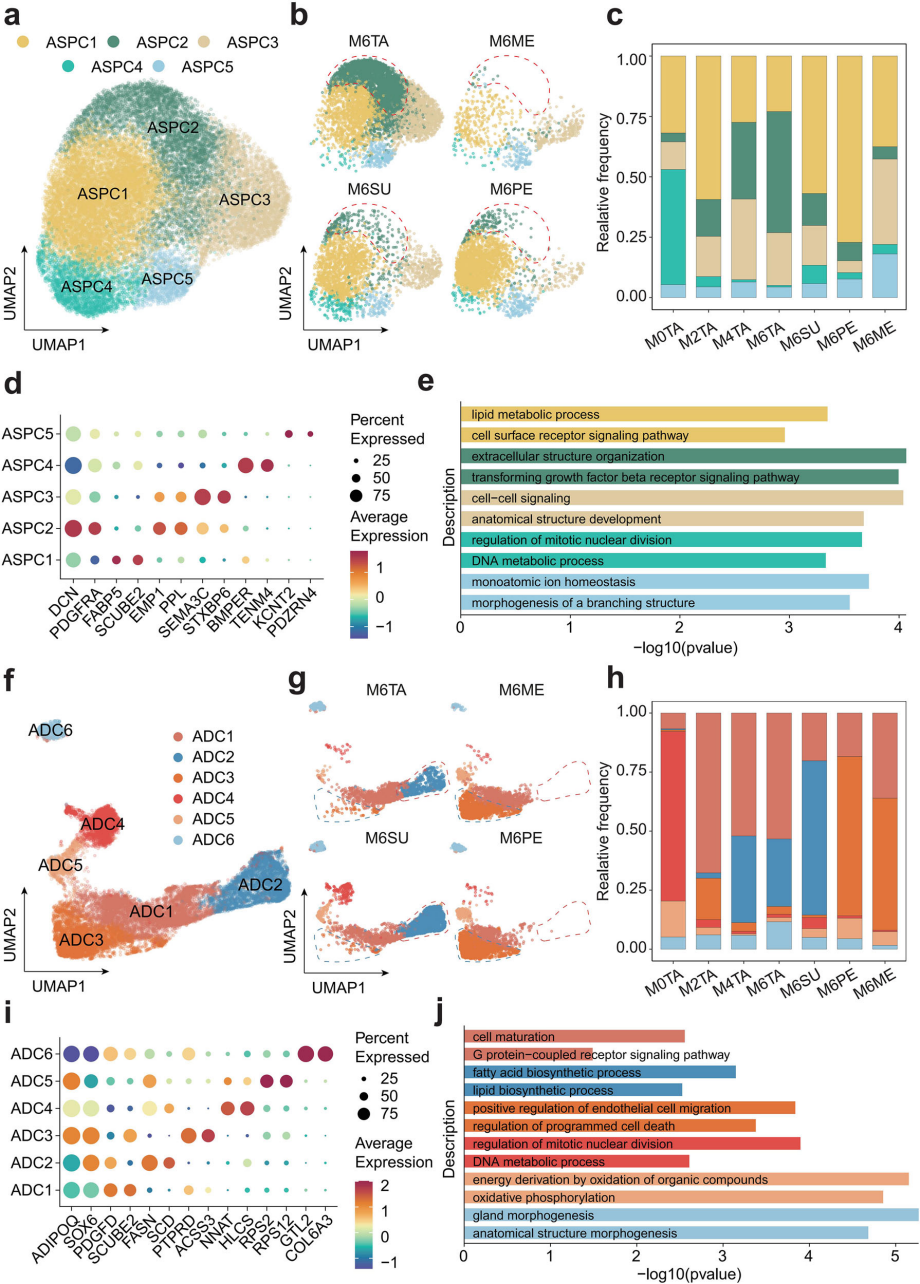
ASPC and ADC exhibit different compositions in different adipose depots. **a** UMAP reclustering of ASPC subpopulations. **b** UMAP clustering of ASPC in four fat depots, with the ASPC2 subpopulation highly enriched in tail fat highlighted by a red dashed line. **c** Proportions of different ASPC subpopulations across the seven groups. **d** Average expression levels of marker genes in each ASPC subpopulation. **e** GO enrichment terms of marker genes for ASPC subpopulations. **f** UMAP reclustering of ADC subpopulations. **g** UMAP clustering of ADC in four fat depots, with the ADC2 subpopulation uniquely present in subcutaneous fat highlighted by a red dashed line, and the ADC3 subpopulation highly enriched in visceral fat highlighted by a blue dashed line. **h** Proportions of different ADC subpopulations across the seven groups. **i** Average expression levels of marker genes in each ADC subpopulation. **j** GO enrichment terms of marker genes for ADC subpopulations. The abbreviations are the same as in Fig. 1.

To explore the cellular heterogeneity of ovine ASPC, we further subdivided the ASPC into five subpopulations (ASPC1-ASPC5) (Fig. 2a). The analysis of cell proportions showed that ASPC1 had a consistently high proportion in adipose tissues at different developmental stages and anatomical locations (Fig. 2c). ASPC1 exhibited high expression of fatty acid-binding protein 5 (*FABP5*), signal peptide, CUB domain and EGF like domain containing 2 (*SCUBE2*), and Fos proto-oncogene, AP-1 transcription factor subunit (*FOS*), which were associated with fatty acid transport and growth development in all fat depots (Fig. 2d and Supplementary Data 4). GO enrichment analysis revealed that ASPC1 was involved with “lipid metabolic processes” and “cell surface receptor signaling pathways,” suggesting that it may be the key ASPC subpopulation driving differentiation in ovine adipose tissues (Fig. 2e). The corresponding ASPC4 subgroup had a higher proportion specifically at 0-day-old. Genes upregulated in ASPC4 included those involved in the BMP pathway, such as BMP binding endothelial regulator (*BMPER*) and bone Morphogenetic Protein 5 (*BMP5*) (Fig. 2d and Supplementary Data 4). GO terms such as “regulation of mitotic nuclear division” and “DNA metabolic process” were also enriched in ASPC4, suggesting that ASPC4 may play an important role in early adipogenic differentiation (Fig. 2e and Supplementary Data 5). Interestingly, ASPC2 was highly enriched specifically in tail fat and exhibited a longitudinal increase in proportion over developmental time (Fig. 2b, c and Supplementary Data 6). Furthermore, the expression of *PDGFRA* in ASPC2 was upregulated, which is a marker of fibroadipogenic progenitor cells with the ability to differentiate into adipocytes or activate fibroblasts^33^. The major upregulated genes included epithelial membrane protein 1 (*EMP1*), S100 calcium binding protein A4 (*S100A4)*, and insulin-like growth factor binding protein 6 (*IGFBP6*), which are associated with pro-fibrotic processes (Fig. 2d and Supplementary Data 4). GO enrichment analysis showed that ASPC2 was associated with “extracellular matrix organization” and the “transforming growth factor-beta receptor signaling pathway,” suggesting that ASPC2 might be involved in the release of pro-fibrotic factors (Fig. 2e). Compared with other fat depots, fibroadipogenic progenitor cells (ASPC2) in tail fat specifically upregulated genes such as *COL1A1* and collagen type I alpha 2 chain (*COL1A2*) that belong to the “Protein digestion and absorption” pathway. This indicates that ASPC2 in tail fat has bidirectional differentiation potential, providing structural support while meeting the tail fat’s need for adipogenic differentiation (Supplementary Fig. S2 and Supplementary Data 7).

At the same time, we also performed subclustering of ADC, resulting in 6 subpopulations (ADC1-ADC6) (Fig. 2f). ADC1 has a certain proportion in different regions and stages, and this subpopulation may be a non-specific major adipocyte (Fig. 2h), with its upregulated genes including *PDGFD*, which is associated with the regulation of sheep fat^24^. In ADC2, genes related to lipid synthesis, such as fatty acid synthase (*FASN*), stearoyl-CoA desaturase (*SCD*), insulin induced gene 1 (*INSIG1*), and ELOVL fatty acid elongase 6 (*ELOVL6*), as well as scavenger receptor class B member 1 (*SCARB1*), which is associated with cholesterol regulation, were significantly upregulated (Fig. 2i and Supplementary Data 8). GO analysis revealed enrichment in “fatty acid biosynthetic process” and “lipid biosynthetic process,” indicating that ADC2 may be the adipocyte subtype responsible for lipid deposition (Fig. 2j and Supplementary Data 9). ADC2 was exclusively present in subcutaneous fat, with the highest proportion observed in dorsal subcutaneous fat, which exhibited a stronger lipid deposition capacity (Fig. 2h and Supplementary Data 10). For example, *SCARB1* is primarily expressed in the subcutaneous fat ADC subpopulation and is significantly upregulated in ADC2 (*p* < 0.00001) (Supplementary Fig. S3c, d). Immunofluorescence results showed that *SCARB1* was only expressed in subcutaneous fat and was absent in visceral fat, consistent with the analysis results (Supplementary Fig. S3e). In contrast, ADC3 was primarily present in visceral fat and highly expressed the genes protein tyrosine phosphatase receptor type D (*PTPRD*) and *FABP5* (Fig. 2g and Supplementary Fig. S3b). *PTPRD* is a key target for fatty liver treatment^34^. It was enriched in “regulation of programmed cell death,” indicating its association with the inflammatory regulation of visceral fat (Fig. 2j). ADC4 was primarily found in 0-day-old ADC and was enriched in the functions “regulation of mitotic nuclear division” and “DNA metabolic process” (Fig. 2j). It is believed to represent a transitional adipocyte subpopulation between ASPC and ADC. Although these cells already express a certain level of mature adipocyte markers, they still retain some characteristics of ASPCs and possess a limited capacity for cell division. ADC5 was present in all samples, with the highest proportion at 0-day-old. It highly expressed ribosomal proteins such as ribosomal protein S2 (*RPS2*), ribosomal protein S12 (*RPS12*), ribosomal protein S19 (*RPS19*), and ribosomal protein lateral stalk subunit P0 (*RPLP0*) (Supplementary Data 8). It was enriched in functions such as “translation,” “peptide biosynthetic process,” and “cytoplasmic translation,” indicating that it had a high protein synthesis capacity (Fig. 2j). These cells might have played a crucial role in lipid metabolism, energy production, and the formation of adipose tissue function. ADC6 was enriched in “collagen metabolic process” and “regulation of developmental process” and highly expressed the preadipocyte cell marker CD34 molecule (*CD34*), suggesting that it may be preadipocytes (Fig. 2j and Supplementary Data 8)^35^. Its proportion was similar across different stages and tissues (Fig. 2h).

To compare the similarities of ASPC and ADC across species, we integrated our defined subpopulations with published human adipose snRNA-seq datasets (Supplementary Fig. S4a)^25^. We performed cross-species mapping between the sheep and human datasets based on homologous genes. The results revealed similarities between ASPC and ADC in sheep and humans (Supplementary Fig. S4b, c). Among these, sheep ADC2 exhibited the highest similarity to human hAd3, and both cell types accounted for a relatively high proportion of subcutaneous adipose tissue in their respective species (Supplementary Fig. S4c)^25^. This indicates that sheep can serve as a research model for studying the regulation of adipose deposition.

### Cellular communication between ASPC and ADC

**Fig. 3:**
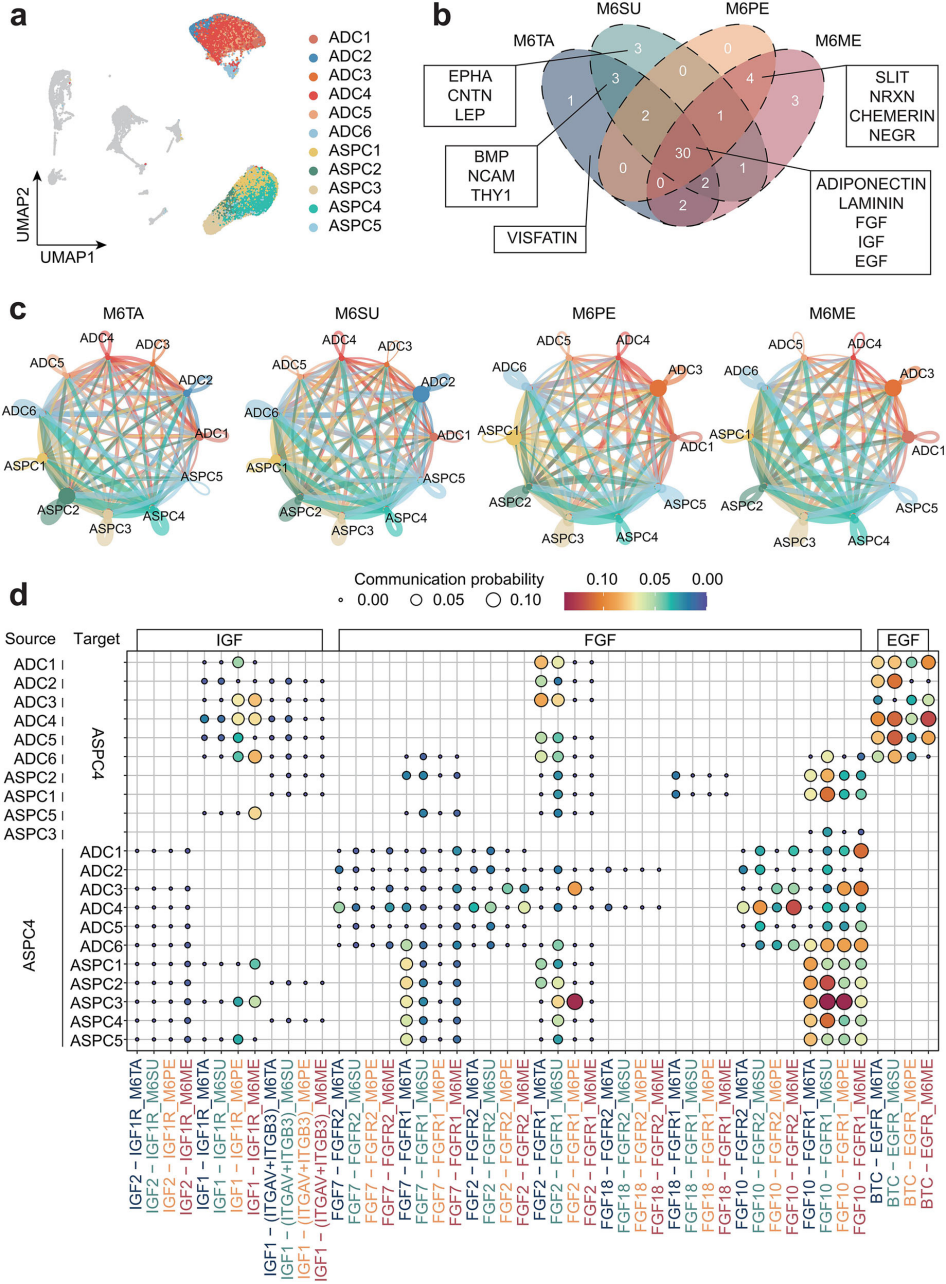
SnRNA-seq revealed distinct cell communication relationships across different fat depots. **a** UMAP of the reclustered ASPC and ADC subpopulations combined. **b** Identification of significant cell communication pathways in the four fat depots, with a Venn diagram showing both shared and tissue-specific communication pathways. **c** Cell-cell communication networks identified as significant across the four fat depots. **d** Heatmap showing the Communication Probability of ASPC4 acting as both sender and receiver for the shared IGF, FGF, and EGF pathways across all tissues. The abbreviations are the same as in Fig. 1.

We identified six ADC subpopulations and five ASPC subpopulations (Fig. 3a). A cell communication analysis was conducted on these eleven subpopulations to determine the interactions between different cell types. Thirty communication pathways were shared among the four types of adipose tissue, including ADIPONECTIN and IGF, which are known to be involved in lipid synthesis, as well as pathways related to FGF and EGF, which are associated with the differentiation and development of adipocytes (Fig. 3b and Supplementary Data 11). In visceral fat, there are four unique pathways, among which CHEMERIN is a novel adipokine associated with obesity and inflammation regulation^36^. Three unique pathways, including BMP, NCAM, and THY1, were identified in subcutaneous fat. The tail fat exhibited a specific pathway known as VISFATIN (Fig. 3b). It is noteworthy that VISFATIN is an adipokine produced and secreted by adipose tissue, which played a role similar to that of insulin and promoted fat synthesis^37^.

Cell communication analysis identified the interactions between subpopulations of adipocytes, many of which involved ASPC4 related to adipocyte differentiation (Fig. 3c). We compared the intensity of outgoing and incoming signals for each cell type, and the results showed that ASPC4 was the main signal emitter in all 4 adipose depots (Supplementary Fig. S5). We quantified the signaling strength of pathways among the 30 tissue-shared cell communication routes in which ASPC4 serves as either the source or the target. The results indicate that ASPC4 is involved in most communication processes and plays distinct roles within them (Supplementary Data 12). Using shared pathways, IGF, FGF, and EGF as examples, the communication relationships between ASPC4 and other subpopulations were demonstrated (Fig. 3d). IGF primarily functions through the *IGF1*-*IGF1R* pathway, showing stronger interaction signals in visceral fat. Insulin-like growth factor 1 (*IGF1*) plays a role in regulating metabolic processes and inflammatory responses^38^. Existing studies have shown that FGF is a critical signal for the differentiation of adipocyte precursors into mature adipocytes^39^. ASPC4 primarily communicates with other ASPC subpopulations through the *FGF10*-*FGFR1* pathway and also exhibits autocrine signaling, suggesting that fibroblast growth factor 10 (*FGF10*) may be an important ligand in this pathway. Additionally, strong *FGF2*-*FGFR1* communication is observed in perirenal fat. EGF exhibits feedback regulation, with its ligand secreted by adipocytes and its receptor secreted by ASPC4. During the process of adipocyte differentiation, various signaling pathways work together to drive the formation of a complex regulatory network, and different adipose tissues possess distinct regulatory pathways.

### Changes in cell communication during fat development

**Fig. 4:**
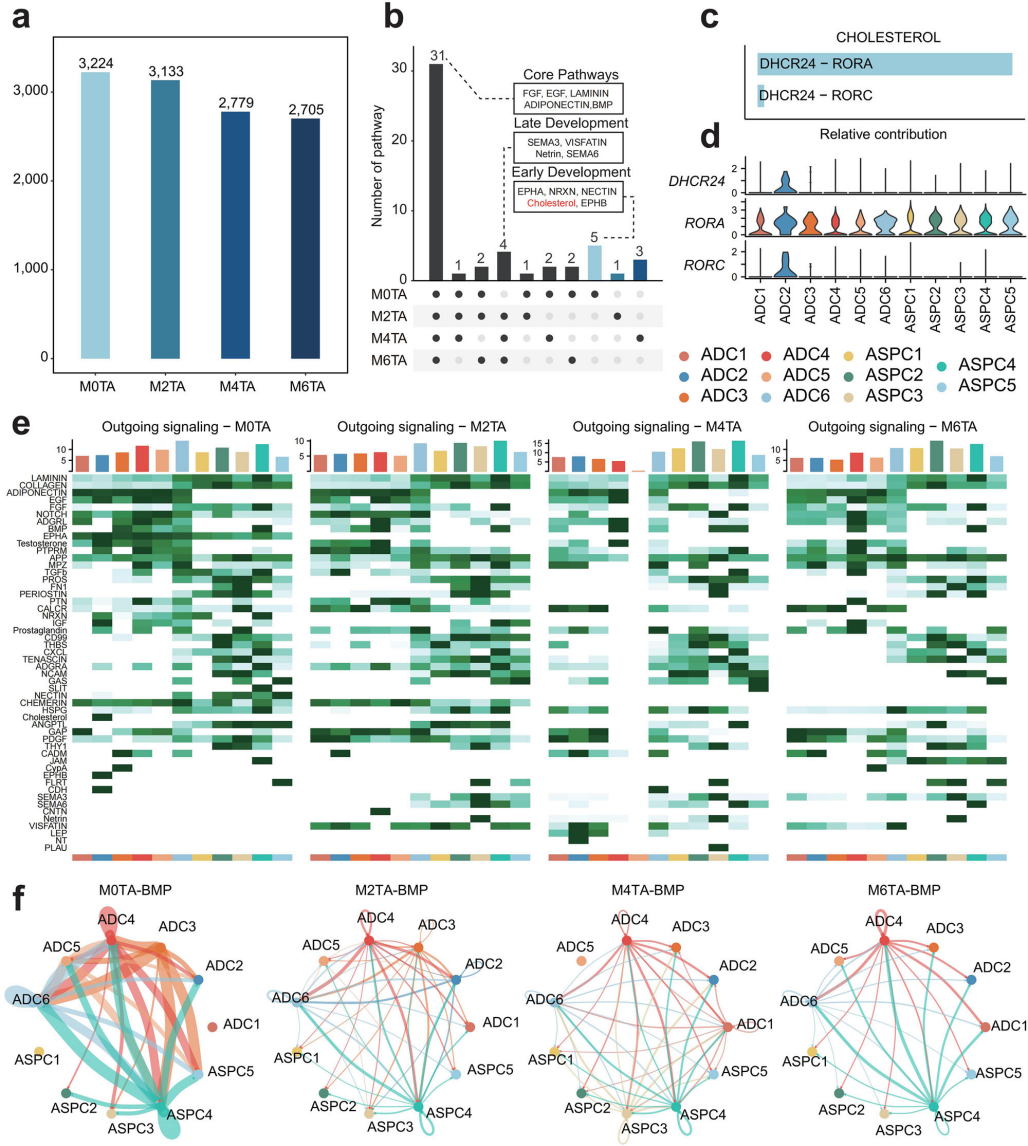
Cellular communication dynamics changed dynamically during longitudinal development. **a** Changes in the total number of cell-cell interactions at different developmental stages. **b** Shared and specific cell signaling pathways at different developmental stages, with the cholesterol pathway highlighted in red, which is an important pathway related to lipid synthesis, specifically present in 0-day-old stages. **c** Contributions of specific ligand-receptor pairs in Cholesterol signaling. **d** Expression levels of receptor and ligand genes involved in the cholesterol pathway across different cell subpopulations. **e** Outgoing signaling of all pathways across the four developmental stages, with the x-axis representing different cell types and the y-axis representing signaling pathways. **f** Changes in the inferred network structure of the BMP pathway across the four developmental stages. The abbreviations are the same as in Fig. 1.

To define the changes in cell communication networks during tail fat development, we performed cell communication analysis using tail fat tissue samples collected at the 0M, 2M, 4M, and 6M stages, constructing dense networks consisting of 3224, 3133, 2779, and 2705 significant cell communication relationships, respectively (Fig. 4a). As the developmental stages progress, the number of interactions gradually decreases, indicating that cell-to-cell interactions are more frequent in the early stages of development. A total of 31 pathways are shared across the four stages, including adipose-shared pathways such as FGF, EGF, LAMININ, and ADIPONECTIN, as well as the BMP pathway specific to subcutaneous fat (Fig. 4b and Supplementary Data 13). It is worth noting that five pathways only appear during early development, including EPHA, NRXN, NECTIN, Cholesterol, and EPHB (Supplementary Data 14). Among them, Cholesterol is a known fat-regulating pathway. In tail fat tissue, Cholesterol primarily acts through 24-dehydrocholesterol reductase (*DHCR24*) as its ligand and RAR Related Orphan Receptor A (*RORA*) as the main receptor (Fig. 4c). The ligand gene is exclusively expressed in ADC2, while the receptor is expressed in both adipocytes and the ASPC subpopulation, which aligns with the feedback regulation mechanism of cholesterol (Fig. 4d)^40^.

Analysis of ligand signals across all 52 pathways revealed that the signaling pathways exhibit dynamic developmental changes (Fig. 4e). ASPC4 and ADC6 were identified as the primary sources of ligands associated with adipocyte differentiation, including ADIPONECTIN, FGF, BMP, TGFβ, COLLAGEN, and EGF. ADC6 is also a major source of receptor signals, indicating that preadipocytes have an active cell communication process (Supplementary Fig. S6). Previous studies have shown that BMP signaling can activate adipocyte transcription factors expressed during development^41^. We identified the differential analysis of 0-day-old and 6-month-old, revealing that the BMP pathway is upregulated in 0-day-old, indicating that the BMP pathway plays a major role in early development (Supplementary Fig. S7a). Therefore, we specifically investigated how BMP-based signaling changes during sheep adipose tissue development. At the 0-day-old stage, ASPC4 is the main source of BMP ligands, while preadipocytes (ADC6) are the primary source of receptors (Supplementary Fig. S7b). The upstream ligand of the BMP pathway is *BMP5*, while the downstream receptors include bone morphogenetic protein receptor type 1A (*BMPR1A*), activin A receptor type 2A (*ACVR2A*), bone morphogenetic protein receptor type 2 (*BMPR2*), and activin A receptor type 1 (*ACVR1*), among which *BMP5* is highly expressed in ASPC4 (Supplementary Fig. S7c, d). BMP signaling showed the highest communication intensity at the M0 stage, which gradually decreased throughout development (Fig. 4f). At different developmental stages, ADC4 and ASPC4 consistently served as the primary sources of ligand signals, exhibiting autocrine activity (Fig. 4f). Ligand *BMP5* is primarily upregulated at 0-day-old (Supplementary Fig. S8a, b). These results suggest that BMP may be a key pathway regulating early adipocyte differentiation, with *BMP5*-*BMPR1A*/*ACVR2A* identified as the main contributor to this communication network.

### Temporal analysis reveals the developmental trajectory of ASPC

**Fig. 5:**
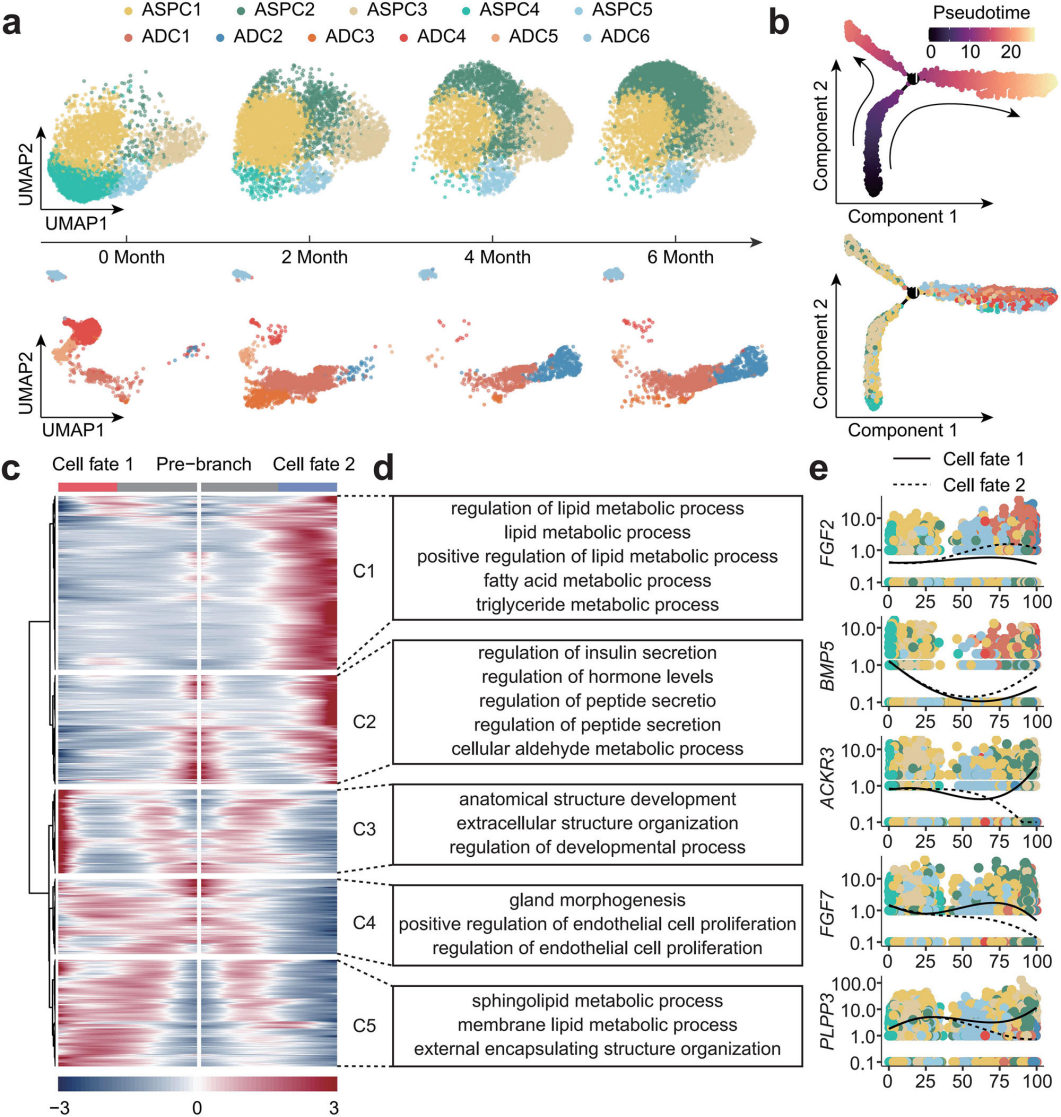
Molecular characteristics of cell differentiation during tail fat development. **a** UMAP clustering changes of ASPC and ADC subpopulations across four developmental stages. **b** Pseudotime trajectory analysis of ASPC and ADC subpopulations using Monocle2, with ASPC4 set as the trajectory starting point. **c** Genes with dynamic expression during tail fat development were clustered into 5 groups based on their expression patterns. **d** GO functional enrichment analysis of genes in each cluster, highlighting significant GO terms (*p* < 0.05). **e** Expression levels of representative genes in each cluster, with colors indicating different cell subpopulations. The abbreviations are the same as in Fig. 1.

The subpopulations of ASPC and ADC in adipose tissue exhibit significant longitudinal dynamic changes (Fig. 5a). Therefore, we performed pseudotime analysis on all subpopulations of ASPC and ADC using the Monocle2 algorithm. By integrating the actual developmental timeline, we identified ASPC4 as the starting point of development. As shown in Fig. 5b, ASPC differentiation follows two distinct fate trajectories: in cell fate 1, the downstream cells remain as ASPCs, while in cell fate 2, the downstream cells differentiate into adipocytes. To explore the molecular determinants of different fate trajectories, we conducted gene expression analyses along the pseudotime trajectory and classified the genes into 5 clusters based on their expression patterns (Fig. 5c). Cluster 1 primarily consists of genes associated with adipocyte characteristics, including elevated expressions of *ADIPOQ*, fibroblast growth factor 2 (*FGF2*), fatty acid-binding protein 4 (*FABP4*), perilipin 1 (*PLIN1*), and *ELOVL6* (Fig. 5e and Supplementary Data 15). These genes are enriched in the categories of “regulation of lipid metabolic process,” “lipid metabolic process,” and “positive regulation of lipid metabolic process” (Fig. 5d). Cluster 2 is primarily expressed early in the trajectory, and interestingly, it also demonstrates high expression downstream of cell fate 2. This cluster includes *BMP5*, an upstream ligand in the BMP pathway, which is categorized among the enriched genes in “regulation of hormone levels” (Fig. 5e). Additionally, the genes in this cluster are also enriched in the categories of “regulation of insulin secretion” and “regulation of peptide secretion,” further suggesting that cluster 2 may play a regulatory role in the differentiation process of adipocytes (Fig. 5d). Cluster 3 is primarily expressed downstream of cell fate 1 and shows upregulation of fibroblast-related genes, including collagen type III alpha 1 chain (*COL3A1*), *DCN*, *COL1A2*, atypical chemokine receptor 3 (*ACKR3*), and collagen type V alpha 2 chain (*COL5A2*) (Supplementary Data 15). The GO enrichment analysis also highlights enrichment in the categories of “anatomical structure morphogenesis” and “extracellular matrix organization,” indicating that Cluster 3 is associated with the structural development of adipose tissue (Fig. 5d). Therefore, we propose that cell fate 1 represents a structural branch. Thus, we successfully described the transcriptional characteristics during the process of adipocyte differentiation and revealed the existence of 2 distinct fate trajectories: the adipogenic branch and the structural branch for ASPC as it differentiates into adipocytes. Notably, preadipocytes (ADC6) are located in the middle of the adipogenic branch trajectory, which is consistent with expectations.

### Differential expression between 0-day-old subcutaneous fat and visceral fat

**Fig. 6:**
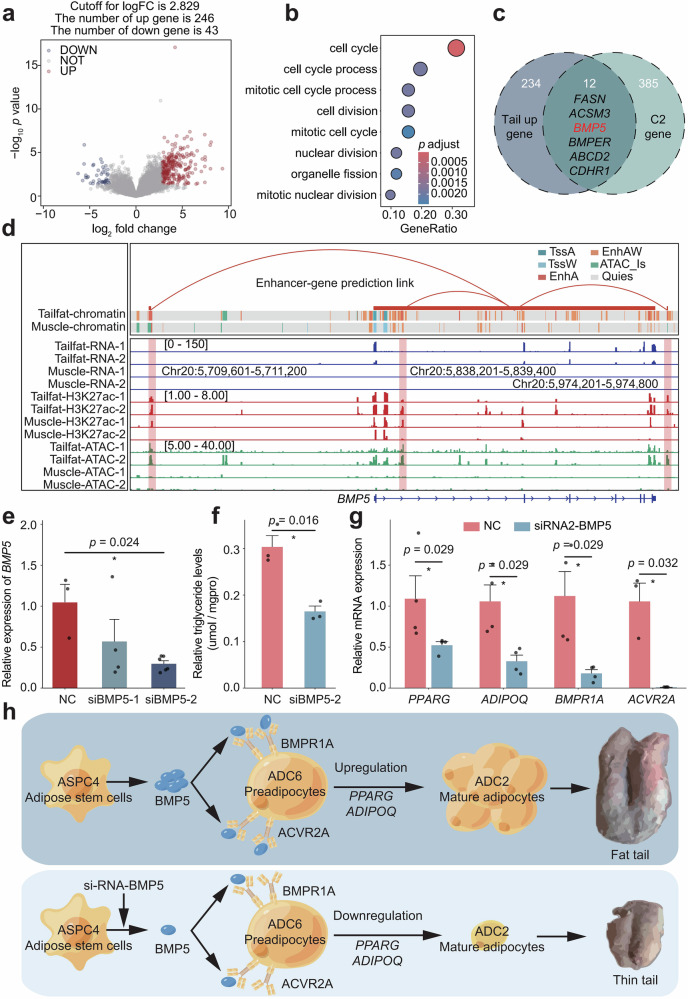
*BMP5* is an important ligand for early subcutaneous fat differentiation. **a** Differentially expressed genes between 0-day-old sheep tail fat and perirenal fat were identified using DESeq2, with “UP” indicating genes upregulated in tail fat and “DOWN” indicating genes downregulated in tail fat. **b** GO functional enrichment analysis of genes upregulated in tail fat. **c** Intersection analysis of tail fat upregulated genes and cluster C2 genes, where *BMP5*, highlighted in red, is identified as the ligand gene specific to the BMP pathway in subcutaneous fat. **d** Published data reveal differences in *BMP5* expression and epigenetic regulation between tail fat and muscle, with the numbers in parentheses representing signal intensity. **e** Interference efficiency after transfecting primary tail adipocytes with BMP5-targeting siRNAs. For *qRT-PCR* experiments, *n* = 6 biological replicates were analyzed. **f** Triglyceride levels in adipocytes transfected with BMP5-siRNA2. Data are presented as mean ± standard error; differences were analyzed using the T-test. *p < 0.05. For triglyceride assays, *n* = 3 biological replicates were used. **g** Expression levels of receptor genes and adipogenic marker genes in adipocytes transfected with BMP5-siRNA2. Data are presented as mean ± standard error; differences were analyzed using the Wilcoxon rank-sum test. *p < 0.05. For *qRT-PCR* experiments, *n* = 4 biological replicates were analyzed. **h** Schematic representation of the proposed mechanism by which *BMP5* regulates adipogenic differentiation.

We compared the bulk transcriptome sequencing data of tail fat and perirenal fat from 0-day-old sheep and analyzed their differentially expressed genes (Fig. 6a). A total of 289 differentially expressed genes were identified, including 246 genes upregulated in tail fat and 43 genes upregulated in perirenal fat (Supplementary Data 16). The genes upregulated in tail fat were mainly enriched in functions related to the cell cycle and mitosis, indicating that tail fat has a greater capacity for differentiation and proliferation (Fig. 6b and Supplementary Data 17). Notably, the genes upregulated in tail fat overlapped with those in Cluster 2, including *BMP5* and its enhancer *BMPER* (Fig. 6c). This is consistent with the results of snRNA-seq, confirming the critical role of the BMP signaling pathway in the early differentiation of subcutaneous fat. Furthermore, *BMP5* can be considered an upstream ligand for further investigation into the regulation of subcutaneous fat deposition.

### Regulatory role of BMP5 in adipogenic differentiation

Based on published data^17^, we first analyzed the expression characteristics of *BMP5* in muscle and tail fat. As shown in Fig. 6d, *BMP5* is exclusively expressed in tail fat, with no expression in muscle, and specific enhancers were also identified in tail fat. This suggests that intramuscular fat differentiation is likely less regulated by *BMP5*. Therefore, we propose that manipulating *BMP5* through gene editing could regulate subcutaneous fat deposition without affecting intramuscular fat deposition. To verify the effect of the *BMP5* gene on subcutaneous fat deposition, we isolated primary adipocytes from tail fat and transfected the cells with small interfering RNA (siRNA) (Fig. 6e and Supplementary Data 18). The results showed that *BMP5* knockdown significantly reduced intracellular triglyceride content (Fig. 6f and Supplementary Data 19), and the expression levels of downstream receptor genes and adipocyte marker genes were also significantly decreased (Fig. 6g and Supplementary Data 20). In summary, the BMP pathway, with *BMP5* as the upstream ligand, was a convincing candidate regulatory pathway influencing subcutaneous adipocyte differentiation.

## Discussion

Currently, single-cell transcriptomics has been extensively employed to characterize adipose tissue in both humans and mice^26,42^. However, research on adipose tissue in sheep is still limited to bulk level analyses^43^. Our study establishes the first spatiotemporal single-cell atlas of sheep adipose tissue, systematically mapping cellular heterogeneity across four anatomically distinct fat depots (dorsal subcutaneous fat, tail fat, perirenal fat, and mesenteric fat) and four developmental stages (0, 2, 4, and 6 months) of the sheep-specific tail fat, including nine distinct cell types such as adipocytes, ASPCs, macrophages etc.

The functional differences between different fat depots are very significant, and they are mainly divided into subcutaneous fat and visceral fat. Among them, visceral fat shows an increased proportion of T cells. T cells accumulate in visceral tissues to maintain systemic metabolic homeostasis and are also related to inflammatory regulation^44^. In contrast, subcutaneous fat has a unique ADC2 associated with lipid deposition, significantly upregulated *FASN* and *SCD* genes were targeted as key enzymes involved in fatty acid synthesis^45^. Subcutaneous fat showed stronger lipid deposition capacity, which is consistent with previous reports^9^. Subcutaneous fat prioritizes lipid storage for heat generation and famine adaptation, whereas visceral fat integrates metabolic and immune homeostasis. We identified specifically highly expressed fibroadipogenic progenitor cells (ASPC2) in the unique sheep fat depot (tail fat). Fibroadipogenic progenitor cells have multiple differentiation potentials, such as adipogenesis, fibrosis, and osteogenesis, and previous reports mainly focused on muscle research^46^. The *PPARGC1B* gene, upregulated in ASPC2, encodes the protein PGC-1β (Supplementary Fig. S3a), which can enhance the activity of the PPAR-γ transcription factor, a regulator of adipocyte differentiation, and plays an important regulatory role in the differentiation of fibro/adipogenic progenitor (FAP) into adipocytes^47^. Tail fat serves as an energy reserve for sheep to withstand harsh environments. We hypothesize that FAPs (ASPC2) may be specifically highly expressed in tail fat to adapt to the demands of energy and lipid storage, supporting the rapid differentiation and repair capacity of tail fat tissue. A unique cell subpopulation ADC3 was also identified in visceral fat, in which many fatty acid metabolism-related genes were upregulated, such as acyl-CoA synthetase short chain family member 3 (*ACSS3*), which is involved in the metabolism of short-chain fatty acids^48^, and *FABP5*, which is related to the metabolism of long-chain fatty acids^49^. In addition, the PPAR signaling pathway was also upregulated in ADC3, but it is mainly mediated by PGC-1α encoded by peroxisome proliferative-activated receptor, gamma, coactivator 1 alpha (*PPARGC1A*) (Supplementary Fig. S3b), which acts on PPAR-α, mainly involved in the oxidation and energy metabolism of fatty acids and has an anti-inflammatory response^50^. ADC3 may be a key adipocyte subset in visceral fat. We also identified a non-specific adipocyte subpopulation ADC1, enriched in the “G protein-coupled receptor signaling pathway,” which in tail fat shows the lowest proportion at 0-day-old, the highest at 2 months, and a similar proportion from 4 to 6 months (Fig. 2f–j). It is also enriched in the cell maturation pathway, mediating the process of transforming cells from an immature state to fully mature and functional cells. Therefore, we believe that 2 months may represent the peak stage of tail fat development. Importantly, we identified a cell subpopulation, ASPC4, with a higher proportion in the early-stage tail fat but a lower proportion across all four fat depots at 6 months. This subpopulation is mainly enriched in functions related to cell division and proliferation and may represent the initial progenitor cells involved in adipocyte differentiation.

Previous reports have shown that adipocyte differentiation is a complex phenotypic trait regulated by multiple pathways^51^. For example, adiponectin is an important adipokine that enhances insulin sensitivity by interacting with its receptors, which triggers the activation of AMPK, PPAR-α, and possibly other signaling pathways^52^. In this study, the adipokine ligand source is identified as adipocytes. Laminin maintains the structural integrity of the basement membrane and influences cell proliferation and differentiation by binding to its receptors^53^. Similarly, the collagen pathway also plays a structural and functional role, being predominantly expressed in FAP, which aligns with our findings. ASPC2 is identified as the primary ligand source^54^. We primarily focused on pathways related to early adipose development. Among them, the BMP pathway is highly active in early adipocytes (0-day-old). Previous studies have shown that the absence of BMP receptors can prevent the formation of adipocytes^41^. BMP belongs to the TGF-β family and is a group of highly conserved functional proteins with similar structures that can stimulate DNA synthesis and cell replication, thereby promoting cell differentiation^55^. BMP signaling plays an important role in the growth and differentiation of cells during early development^56^. *BMP2*, bone morphogenetic protein 4 (*BMP4*), and bone morphogenetic protein 7 (*BMP7*) can all induce intracellular lipid accumulation and PPARγ expression. While *BMP2* and *BMP4* primarily promote the formation of white adipose tissue, *BMP7* enhances PR/SET domain 16 (*PRDM16*) expression to facilitate brown adipose tissue formation^57,58^. Long et al. reported that bta-miR-493 promoted preadipocyte proliferation by targeting *BMPR1A* through the BMP and p38MAPK signaling pathways^59^. Our results show that there are four possible BMP pathways in early adipose differentiation, with *BMP5* as the ligand and ASPC4 as the main ligand source, and it only acts in subcutaneous fat. *BMP5* plays a functional role in the regulation of hepcidin and iron homeostasis^60^, and iron storage is related to adipose tissue and lipid metabolism^61^. Studies have also shown that QTLs in *BMP5* are associated with fat deposition traits in pigs^62^. Our functional validation results confirmed that inhibition of *BMP5* expression suppresses adipogenesis, which further corroborated previous reports.

Although the process of adipocyte progenitor cell differentiation into adipocytes has been widely described^51,63^, a comprehensive characterization of adipocyte dynamics during tail fat development is still lacking. Therefore, we conducted a developmental trajectory analysis of ASPC and ADC. We observed two fate trajectories during the differentiation of ASPC into adipocytes, namely the adipogenic branch and the structural branch. In the late stages of the adipogenic fate trajectory, functions related to lipid metabolism are primarily enriched, whereas in the structural branch, functions related to structural morphogenesis are predominantly enriched. ASPC usually contains adipose stem cells and regulatory progenitor cells, among which adipose stem cells can eventually differentiate into adipocytes^64^, similar to our results. Based on gene expression patterns, we identified Cluster 2 as the regulatory genes for adipocyte differentiation, including *BMP5*, lipoprotein lipase (*LPL*), and encoding enoyl-CoA hydratase/L-3-hydroxyacyl-CoA dehydrogenase (*EHHADH*) (Supplementary Data 15). *EHHADH* plays a role in fatty acid oxidation and metabolism, while *LPL* is directly involved in fat metabolism^65^. Our results suggest that the genes in Cluster 2 can serve as key genes for the regulation of fat deposition.

In summary, our study identified the cellular heterogeneity and functional differences across four fat depots in sheep and characterized the longitudinal developmental changes in tail fat. Unique cell subpopulations and regulatory pathways with distinct roles in different fat depots were revealed. Based on the actual developmental timeline, the differentiation trajectory of adipocytes was explored. This resource can help identify potential candidate genes regulating fat deposition traits in sheep. Notably, the subcutaneous fat-specific BMP signaling pathway (*BMP5*-*BMPR1A*/*ACVR2A*) identified through screening could be of great interest. This result provides valuable candidate genes that could facilitate the breeding of low-fat sheep through genomic selection or gene editing (Fig. 6h). This study also has certain limitations. First, although the longitudinal developmental changes of fat were characterized based on tail fat, it may not fully cover the developmental changes of all fat depots. Second, although we identified *BMP5* as a candidate gene regulating sheep fat differentiation, the process of adipocyte differentiation is complex, and further exploration of other regulatory pathways is needed to evaluate the comprehensive regulatory network of adipocyte differentiation. In subsequent studies, additional adipose regulatory pathways can be screened using our atlas, and the identified candidate genes can be applied to production practices via gene editing technology, reducing excessive fat deposition and improving feed energy utilization efficiency. This aligns with the development goals of sustainable, low-cost, and high-efficiency animal husbandry.

## Methods

### Animals and sample collection

Male Hu sheep were used as the research object. Dorsal Subcutaneous fat, tail fat, perirenal fat, and mesenteric fat were collected from the same healthy adult (6 months) Hu sheep. Additionally, tail fat was collected at the newborn (0-day-old), weaning (2 months), and puberty (4 months) stages. After the experimental sheep were euthanized, fat tissues were immediately isolated, washed with pre-cooled phosphate-buffered saline (PBS) to remove blood, and preserved in liquid nitrogen.

### Single-cell nucleus extraction and library construction

Frozen adipose tissue was homogenized in 3 ml of lysis buffer (10 mM Tris (pH 7.4), 10 mM NaCl, 3 mM MgCl_2_, and 0.05% (v/v) NP-40 detergent) using a glass dounce homogenizer. The sample was then incubated in 5 ml of lysis buffer for 5 min for lysis, followed by the addition of 5 ml of wash buffer (10 mM Tris (pH 7.4), 10 mM NaCl, 3 mM MgCl_2_, 1% BSA, 1 mM DTT, RNase inhibitor 1 U/µl, nuclease-free water). The sample was filtered through a 30 µm cell strainer and centrifuged at 500 × *g* for 5 min. After centrifugation, the nuclei pellet was resuspended in 5–10 ml of wash buffer by gently pipetting up and down 8–10 times to ensure proper dispersion. The suspension was washed 3 times, and the nuclei were then resuspended in 1 ml of wash buffer before being mixed with 25% Optiprep. The mixture was layered onto a 29% Optiprep cushion and centrifuged at 10,000 × *g* for 30 min. Finally, the nuclei pellet was resuspended in wash buffer, with an additional 3 washes to ensure purity.

The isolated nuclei were assessed for quality using AO/PI staining (via a LUNA counter) and observed under a microscope for nuclear integrity (Supplementary Fig. S9). The purified nuclei were then resuspended in Nuclei Resuspension Buffer (Nuclei buffer (10X Genomics, 20x) 1x, 1 mM DTT, RNase inhibitor 1 U/μl, Nuclease-free Water) at a final concentration of approximately 1 × 10^6^ nuclei/ml.

The snRNA-seq libraries were prepared according to the manufacturer’s protocol (10X Genomics) and sequenced on a NovaSeq 6000 platform (Illumina, California, USA) using paired-end 150 bp (PE150) sequencing strategy.

### Bulk RNA library construction and sequencing

For bulk RNA-seq, total RNA was extracted from tissues using TRIzol reagent (Invitrogen). A total of 4 biological replicates of tail fat and perirenal fat from 0-month-old Hu sheep were included in the study. Library construction was performed using the VAHTS Total RNA-seq Library Prep Kit for Illumina® (Vazyme) following the manufacturer’s instructions. After library construction, preliminary quantification was conducted with a Qubit 3.0 Fluorometer, and libraries were diluted to a concentration of 1 ng/μL. Subsequently, library quality was assessed using an Agilent 2100 Bioanalyzer, followed by accurate quantification of the effective library concentration (with a requirement of effective concentration > 10 nM). Finally, high-throughput sequencing was carried out on an Illumina NovaSeq 6000 platform.

### SnRNA-seq data analysis

For snRNA-seq, we used the cellranger mkref function of the 10X Genomics Cell Ranger (version 7.2.0) pipeline to construct the sheep reference genome (Oar_rambouillet_v1.0 (GCF_002742125.1), downloaded from https://ftp.ncbi.nlm.nih.gov/genomes/all/GCF/002/742/125/GCF_002742125.1_Oar_rambouillet_v1.0/GCF_002742125.1_Oar_rambouillet_v1.0_genomic.fna.gz). We used the cellranger (version 7.2.0) count function to process fastq files, filter reads, align, perform technical analysis, and generate matrix files for quantitative analysis^66^. We performed downstream analysis using the Seurat package (version 5.0.3) in R (version 4.3.3)^67^. We filtered the raw cells with the following criteria: 500 < number of gene expressions < 4000, mitochondrial gene proportion < 5%. Data normalization was performed using the NormalizeData() function with the parameters “normalization.method = “LogNormalize,” scale.factor = 10,000.” Highly variable genes were identified using the FindVariableFeatures() function. Dimensionality reduction was performed using the RunPCA() function with the top 50 principal components selected. Batch effect correction for 7 groups was carried out using Harmony (version 1.2.0)^68^. Cell clustering analysis was performed using the FindClusters() function with the resolution parameter set to 0.1, identifying 9 clusters. Uniform Manifold Approximation and Projection (UMAP) was performed using the RunUMAP() function. Differentially expressed genes in cells were identified using the FindAllMarkers() function. Gene Ontology (GO) analysis of marker genes was conducted using the clusterProfiler package (version 4.10.1)^69^. Differentially expressed genes between groups were screened using the FindMarkers() function. Gene Set Enrichment Analysis (GSEA) was performed using the clusterProfiler package (version 4.10.1).

### Cross-species comparisons between humans and sheep

We downloaded human ASPC and ADC datasets from the Single Cell Portal (study no. SCP1376)^25^. Homologous genes between sheep and humans were identified using BioMart. We then retained only the homologous genes present in both the human and sheep expression matrices, followed by a similarity analysis using the MetaNeighbor package (version 1.22.0)^70^.

### Cell communication analysis

Cell communication analysis was conducted using the CellChat package (version 2.1.2)^71^ in R (version 4.3.3). Due to the absence of complete ligand-receptor annotations in the sheep genome, homologous human genes were used for subsequent analysis. First, an object was constructed using the createCellChat() function. Communication probabilities were inferred using the computeCommunProb() function, and filtering was performed using the filterCommunication() function with the parameter “min.cells = 10.” The interaction network was integrated using the aggregateNet() function. Visualization was carried out using the built-in functions of the CellChat package (version 2.1.2).

### Pseudotime trajectory analysis

Pseudotime analysis was performed using the Monocle package (version 2.30.1)^72^. The analysis object was constructed using the newCellDataSet() function, and genes were ordered based on differential expression. Dimensionality reduction was performed using the reduceDimension() function with the additional parameters “method = ‘DDRTree’, dist.class = ‘spam::dist’.” ASPC4 was determined as the starting point of the trajectory based on the true temporal order.

### Bulk RNA-seq data analysis

For bulk RNA-seq data, fastp (version 0.23.4)^73^ was used to process raw reads, remove adapters, and trim low-quality bases, with the supplementary parameter “--detect_adapter_for_pe.” Clean reads were aligned to the sheep reference genome (Oar_rambouillet_v1.0 (GCF_002742125.1), downloaded from https://ftp.ncbi.nlm.nih.gov/genomes/all/GCF/002/742/125/GCF_002742125.1_Oar_rambouillet_v1.0/GCF_002742125.1_Oar_rambouillet_v1.0_genomic.fna.gz) using hisat2 (version 2.2.1)^74^. The aligned reads were sorted and converted into BAM files using Samtools (version 1.6)^75^. FeatureCounts (version 2.0.3) was used to quantify the transcripts of each gene for subsequent analysis. Differential gene analysis was performed using the DESeq2 package (version 1.42.0) (*p* < 0.05). Gene Ontology (GO) analysis of differential genes was conducted using the clusterProfiler package (version 4.10.1)^69^.

### Immunofluorescence (IF) staining

Paraffin-embedded wax blocks were sectioned and deparaffinized. The sections were incubated in 3% hydrogen peroxide solution for 25 min and rinsed in PBS. Afterward, the sections were blocked with bovine serum albumin (BSA) (Wuhan Saiweier Biotechnology Co., Ltd., China) for 30 min, followed by incubation with the primary antibody (Wuhan Saiweier Biotechnology Co., Ltd., China) overnight. The HRP-conjugated secondary antibody was then added, and the sections were incubated at room temperature for 50 min. TSA dye was applied and incubated in the dark for 10 min. The nuclei were counterstained with DAPI (Wuhan Saiweier Biotechnology Co., Ltd., China) at room temperature for 10 min. Anti-fluorescence quenching mounting medium was used for sealing. Finally, the sections were observed using CaseViewer software (version 2.4.0).

### BMP5 siRNA interference

Cells were isolated and cultured according to previously published protocols^17^. In short, preadipocytes were isolated from tail adipose tissues of 10-day-old Hu sheep. Tail fat specimens were diced into roughly 1-mm^3^ cubes and finely minced. The tissue pieces were then digested with type I collagenase to isolate preadipocytes, which were cultured in basal medium supplemented with 10% fetal bovine serum (FBS; Hyclone) and 1% antibiotic-antimycotic mixture. Cell suspensions were plated into 25-cm^2^ culture flasks and incubated at 37 °C in a 5% CO_2_ environment. The culture medium was changed after the initial 24 h, and then refreshed every 48 h thereafter. Once primary preadipocytes reached about 80% confluency, they were trypsinized and subcultured at a 1:3 ratio. The third passage of these cells was used for all subsequent experimental analyses. Two pairs of siRNA targeting *BMP5* were synthesized by GenePharma Co., Ltd. (Shanghai, China), with sequence information provided in the Supplementary Data 21. The si-BMP5-2 sequence was chosen for further analysis due to its efficiency. Sheep preadipocytes from tail fat were passaged into 12-well plates, and when the cell density reached 80–90%, siRNA transfection was performed using jetPRIME transfection reagent. After 6 h, the medium was replaced with induction medium containing 10% FBS, 0.5 mM 3-isobutyl-1-methylxanthine, 0.02 mM rosiglitazone, 0.0255 mM dexamethasone, and 0.01 mg/ml insulin. Cells were collected after 48 h for RNA extraction.

### RNA extraction and quantitative real-time reverse transcription PCR (qRT-PCR)

Total RNA was extracted from cells using the TRIzol reagent, and RNA concentration was measured with a NanoDrop 2000 spectrophotometer (Thermo Fisher Scientific, Massachusetts, USA). Reverse transcription was performed with the Evo M-MLV RT Premix Kit (Accurate Biotechnology, Hunan, China) according to the manufacturer’s instructions. Primers for qRT-PCR were designed using the National Center for Biotechnology Information database, with detailed sequences provided in the Supplementary Data 21. The reactions were performed using NovoStart® SYBR High-Sensitivity qPCR SuperMix (Novoprotein, Suzhou, China) according to the manufacturer’s recommended protocol. *UXT* was used as the reference gene, and relative gene expression levels were calculated using the 2^−ΔΔCt^ method.

### Triglyceride detection

Sheep preadipocytes were transfected with siRNA and induced to differentiate for 48 h using a differentiation medium. After induction, cells were washed three times with ice-cold PBS. Triglyceride levels were then measured using a triglyceride assay kit (APPLYGEN, Beijing, China) according to the manufacturer’s instructions after cell lysis.

### Ethics approval and consent to participate

In this study, all experimental procedures and sample collection associated with the sheep were approved by the Animal Welfare and Ethics Committee of Lanzhou University (No.: 2020-01 and 2021-02). We have complied with all relevant ethical regulations for animal use.

The flowchart was created using Figdraw (authorization code: YIUSI6f3ff).

### Statistics and reproducibility

Statistical analyses were performed using R software (version 4.3.3), with specific methodologies detailed in the Methods section. Cell marker genes were identified using the FindAllMarkers function in Seurat, with the built-in Wilcoxon test for statistical analysis and the Benjamini-Hochberg method for *p* value correction. Differentially expressed genes (DEGs) in bulk RNA-seq data were identified using a combined threshold of *p* < 0.05 and abs(log_2_FoldChange) > 2.829(mean(abs(log_2_FoldChange)) + 2 * sd(abs(log_2_FoldChange))). Significant functional pathways were defined by a *p* value < 0.05. For qRT-PCR and triglyceride detection, a minimum of three biological replicates were used for each group.

## Supplementary information

Description of Additional Supplementary Files

Supplementary Information

Supplementary Data

## Data Availability

The datasets supporting the conclusions of this article are available in the SRA repository (SnRNA-seq and bulk RNA-seq: PRJNA1255873). The numerical Source data underlying all graphs in the manuscript can be found in the Supplementary Data file.

## Code availability

The code used in this study is available on GitHub (https://github.com/Jiangbo-Cheng/Ovis-fat-single-cell-atlas).
