# Supplementary material for: Single-cell atlas reveals cellular heterogeneity and BMP5-mediated regulation of adipogenic differentiation in sheep adipose tissue
Source: Commun Biol. 2026 Jan 21;9:292. doi: 10.1038/s42003-026-09581-3 (PMC12923642; doi:10.1038/s42003-026-09581-3)
Supplement: Supplementary file 1 — Description of Additional Supplementary Files [file 42003_2026_9581_MOESM1_ESM.docx]

File name: Supplementary Data 1

Description: The source data for Supplementary Figure 1b.

File name: Supplementary Data 2

Description: The source data for Figure 1c and Supplementary Figure 1c.

File name: Supplementary Data 3

Description: The source data for Figure 1d.

File name: Supplementary Data 4

Description: The source data for Figure 2d.

File name: Supplementary Data 5

Description: The source data for Figure 2e.

File name: Supplementary Data 6

Description: The source data for Figure 2c.

File name: Supplementary Data 7

Description: The source data for Supplementary Figure 2.

File name: Supplementary Data 8

Description: The source data for Figure 2i and Supplementary Figure 3.

File name: Supplementary Data 9

Description: The source data for Figure 2j.

File name: Supplementary Data 10

Description: The source data for Figure 2h.

File name: Supplementary Data 11

Description: The source data for Figure 3b.

File name: Supplementary Data 12

Description: The source data for Figure 3d.

File name: Supplementary Data 13

Description: The source data for Figure 4b.

File name: Supplementary Data 14

Description: All cellular communication pathways inferred in M0TA.

File name: Supplementary Data 15

Description: The source data for Figure 5.

File name: Supplementary Data 16

Description: The source data for Figure 6a.

File name: Supplementary Data 17

Description: The source data for Figure 6b.

File name: Supplementary Data 18

Description: The source data for Figure 6e.

File name: Supplementary Data 19

Description: The source data for Figure 6f.

File name: Supplementary Data 20

Description: The source data for Figure 6g.

File name: Supplementary Data 21

Description: Details of primer sequences used for qRT-PCR and small interfering RNAs (siRNAs).
