# Supplementary material for: Single-cell atlas reveals cellular heterogeneity and BMP5-mediated regulation of adipogenic differentiation in sheep adipose tissue
Source: Commun Biol. 2026 Jan 21;9:292. doi: 10.1038/s42003-026-09581-3 (PMC12923642; doi:10.1038/s42003-026-09581-3)
Supplement: Supplementary file 2 — Supplementary Information [file 42003_2026_9581_MOESM2_ESM.pdf]

Single-Cell Atlas Reveals Cellular Heterogeneity and *BMP5*-Mediated Regulation of Adipogenic Differentiation in Sheep Adipose Tissue

Jiangbo Cheng[a], Kunchao Han[a], Dan Xu[a], Huibin Tian[a], Xiaoxue Zhang[b], Liming Zhao[a], Xiaobin Yang[a], Deyin Zhang[a], Kai Huang[a], Yukun Zhang[a], Yuan Zhao[a], Xiaolong Li[a], Quanzhong Xu[a], Zongwu Ma[a], Weiwei Wu[c], Jianlin Wang[a], Fadi Li[a], Weimin Wang[a]*

[a]State Key Laboratory of Herbage Improvement and Grassland Agro-ecosystems; Key Laboratory of Grassland Livestock Industry Innovation, Ministry of Agriculture and Rural Afairs; Engineering Research Center of Grassland Industry, Ministry of Education; College of Pastoral Agriculture Science and Technology, Lanzhou University, Lanzhou 730020, China

[b]College of Animal Science and Technology, Gansu Agricultural University, Lanzhou 730070, China

[c]Xinjiang Academy of Animal Sciences, Urumqi, Xinjiang 830011, China

*Corresponding author at: College of Grassland Agriculture Science and Technology, Lanzhou University, 222 Tianshui South Road, Chengguan District, Lanzhou, Gansu, China.

E-mail address: Weimin Wang, wangweimin@lzu.edu.cn

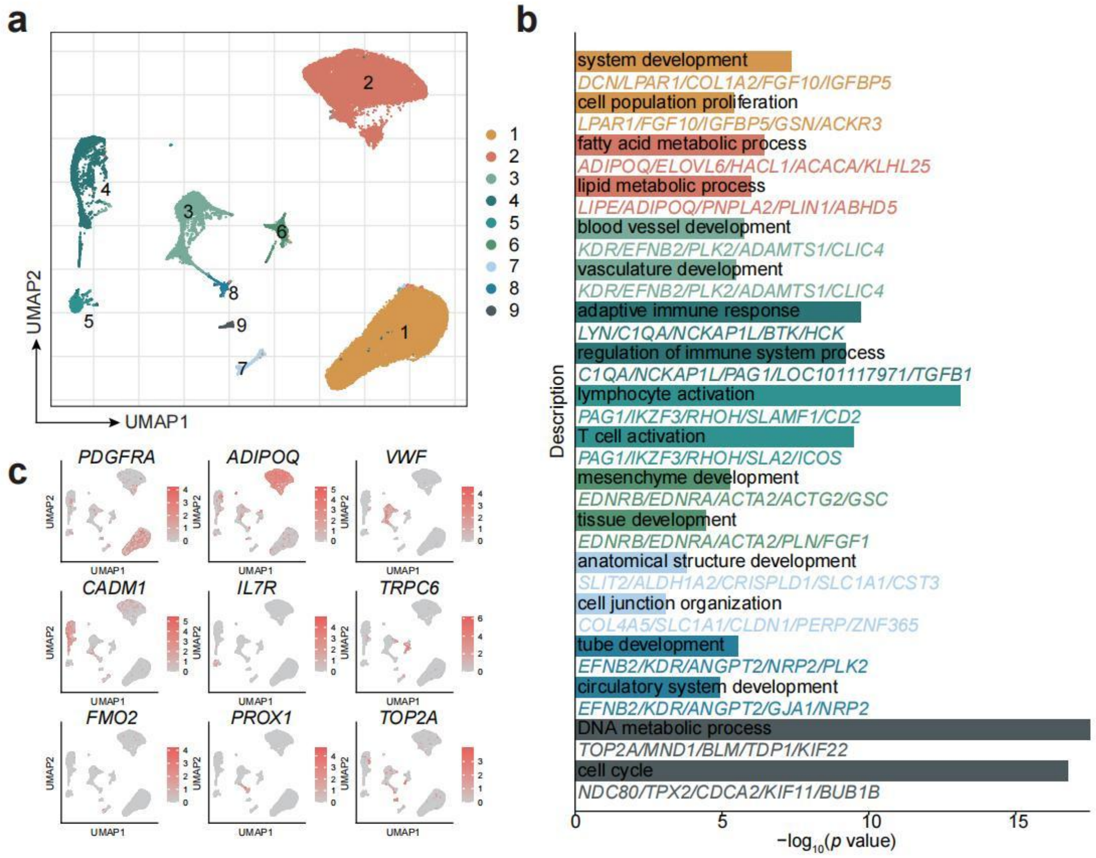

Supplementary Figure 1. (a) Unsupervised clustering revealed nine cell clusters, which were projected onto a UMAP. (b) GO enrichment terms of marker genes for different cell clusters and the top five genes in each term. (c) Expression patterns of marker genes on the UMAP.

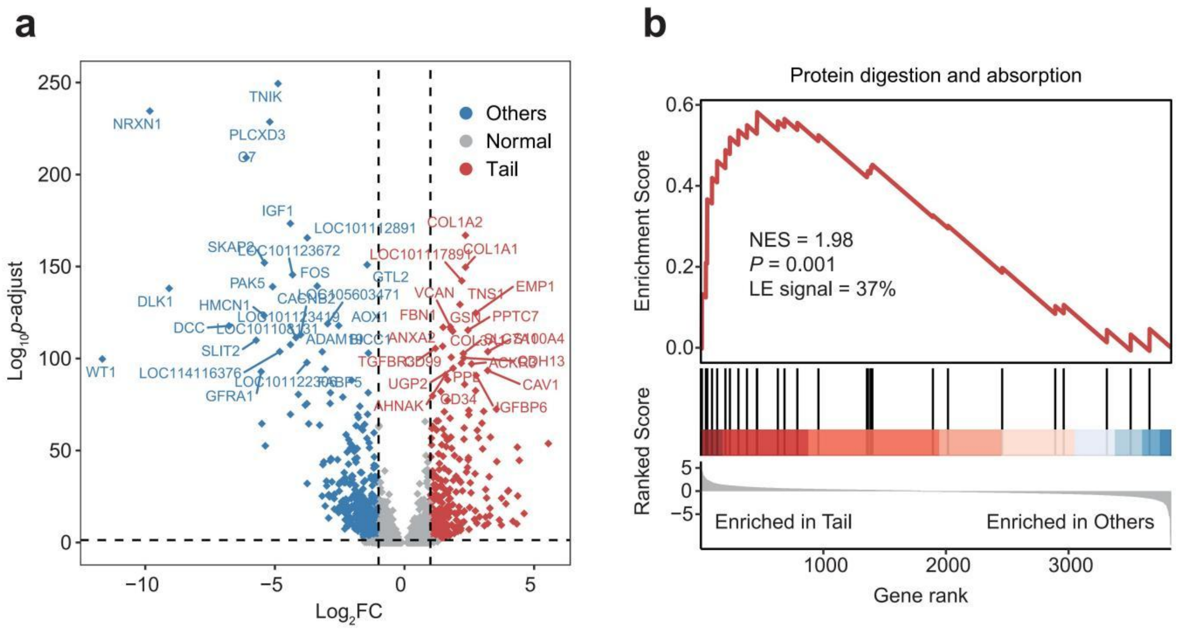

Supplementary Figure 2. (a) Differentially expressed genes within ASPC2 between tail fat and other fat depots were identified using the FindMarkers() function. "Tail" denotes genes upregulated in tail fat, while "Others" denotes genes downregulated in tail fat. (b) Gene set enrichment analysis (GSEA) of KEGG pathways was performed using all genes ranked by expression changes.

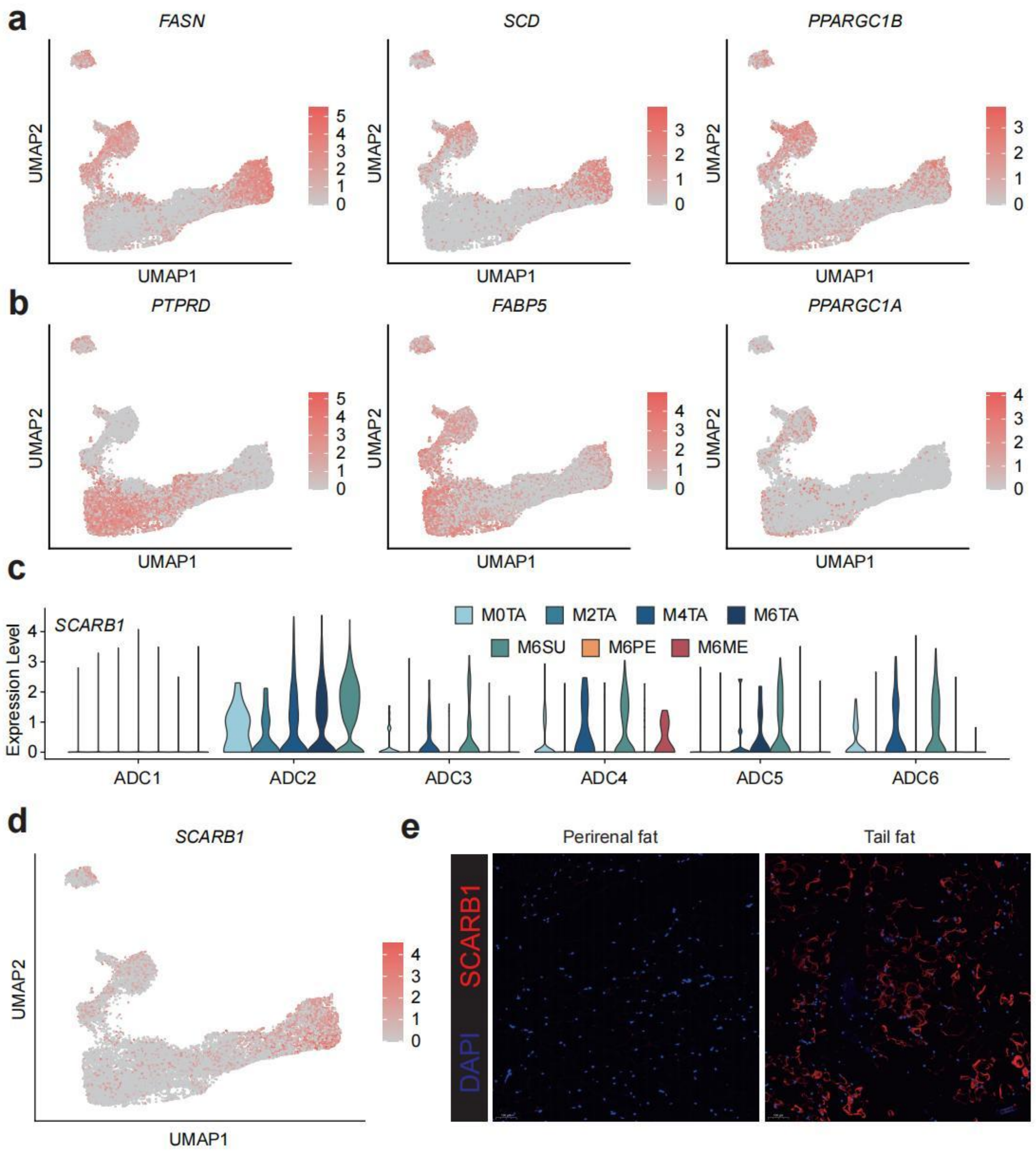

Supplementary Figure 3. (a) Expression patterns of marker genes in ADC2 on the UMAP. (b) Expression patterns of marker genes in ADC3 on the UMAP. (c) Expression levels of the ADC2 marker gene *SCARB1* across different groups. (d) Expression patterns of *SCARB1* on the UMAP. (e) Identification of ADC2 in visceral and subcutaneous fat using *SCARB1* immunofluorescence staining. Scale bar represents 100 μm. The abbreviations are the same as in Fig. 1.

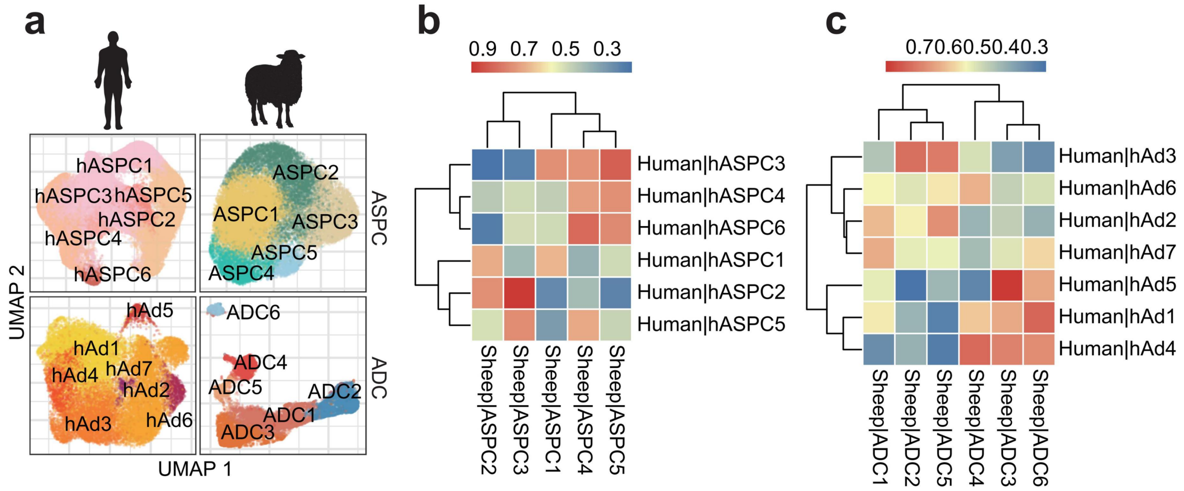

Supplementary Figure 4. (a) UMAP plots of ASPC and ADC subpopulations in humans and sheep. (b) Reproducibility analysis of ASPC clusters between the two species. (c) Reproducibility analysis of ADC clusters between the two species. The heatmap colors correspond to AUROC values, which indicate similarity.

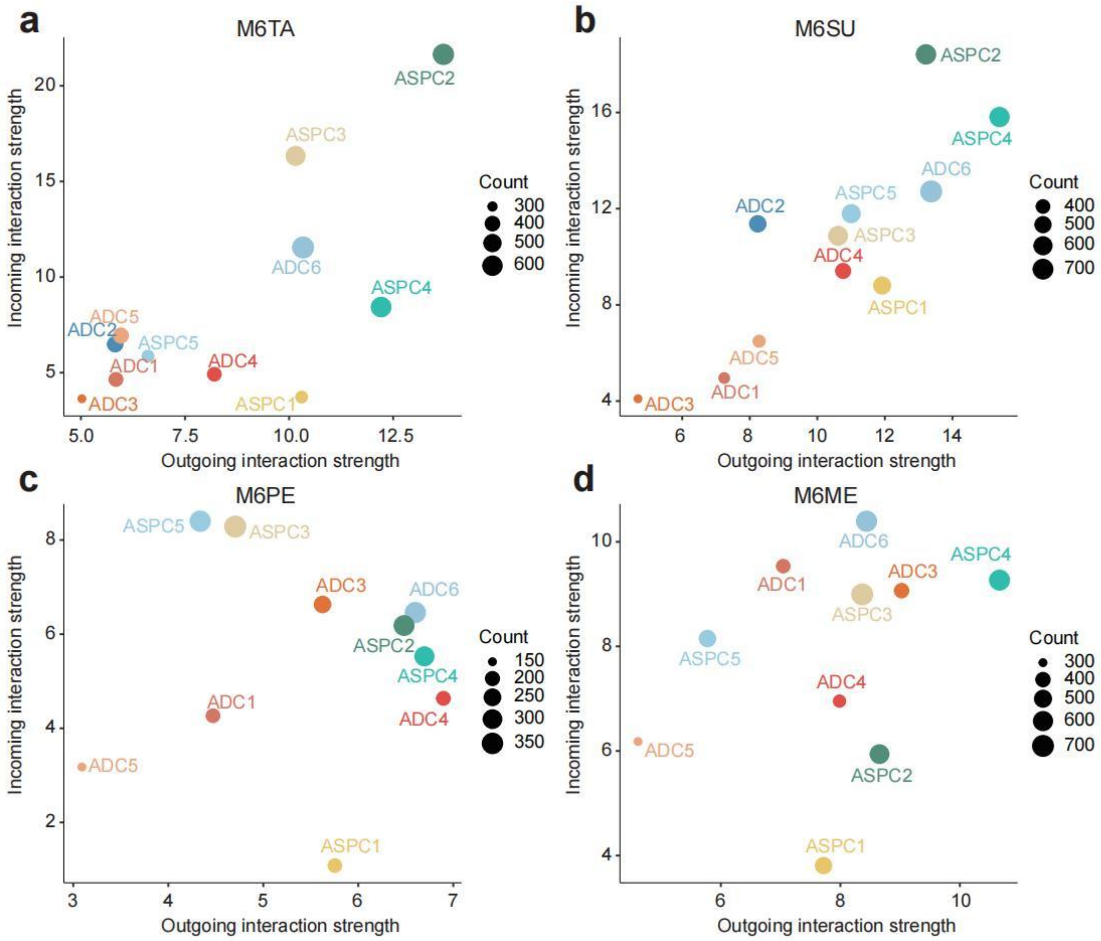

Supplementary Figure 5. Scatter plots showing the signaling strength of each cell type as senders and receivers in each fat depot: (a) M6TA; (b) M6SU; (c) M6PE; (d) M6ME. The abbreviations are the same as in Fig. 1.

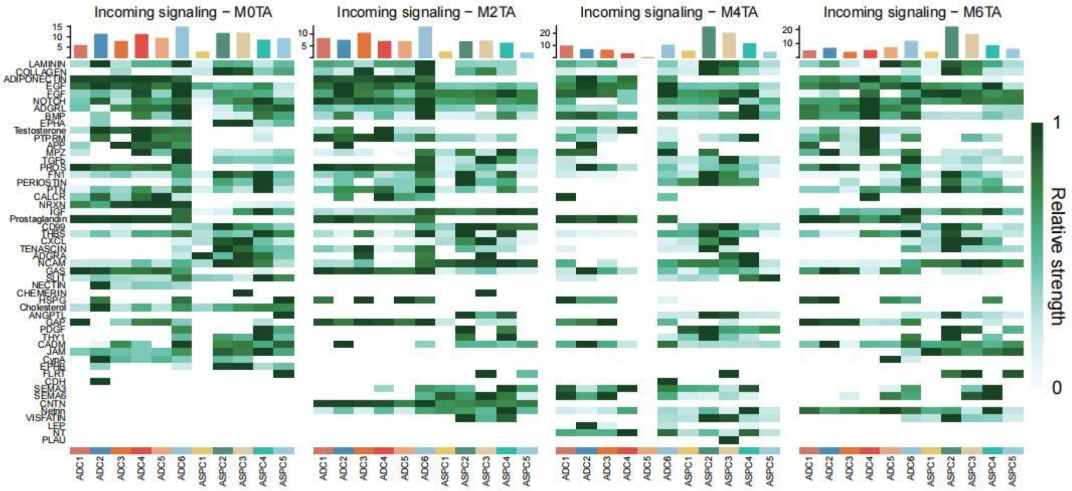

Supplementary Figure 6. Incoming signaling of all pathways across the four developmental stages, with the x-axis representing different cell types and the y-axis representing signaling pathways. The abbreviations are the same as in Fig. 1.

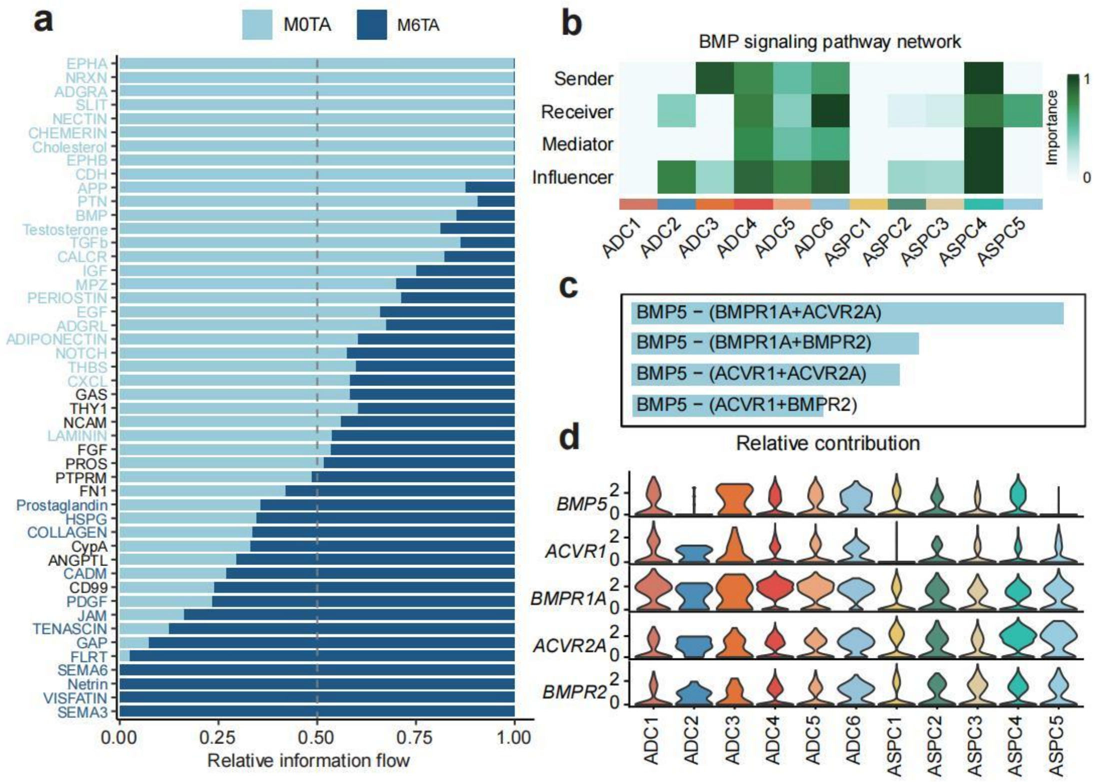

Supplementary Figure 7. (a) Differential pathway analysis of M0TA and M6TA based on rankNet, with black indicating non-differential pathways. (b) Heatmap showing the roles of different cell subpopulations in BMP signaling at the M0TA stage. (c) Contributions of specific ligand-receptor pairs in BMP signaling. (d) Expression patterns of BMP signaling ligand and receptor genes in M0TA. The abbreviations are the same as in Fig. 1.

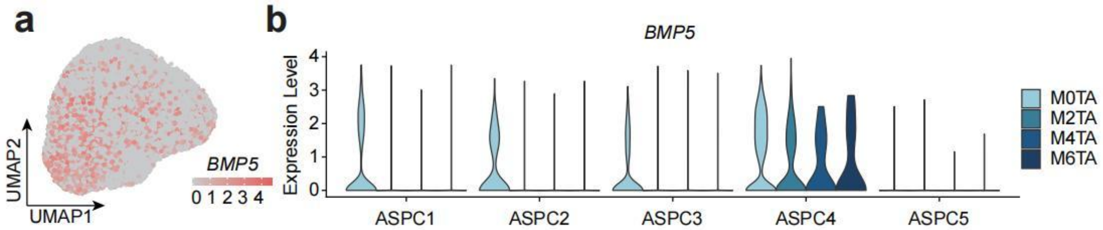

Supplementary Figure 8. (a) Expression pattern of *BMP5* in ASPC on the UMAP. (b) Expression levels of *BMP5* across the 4 developmental stages.

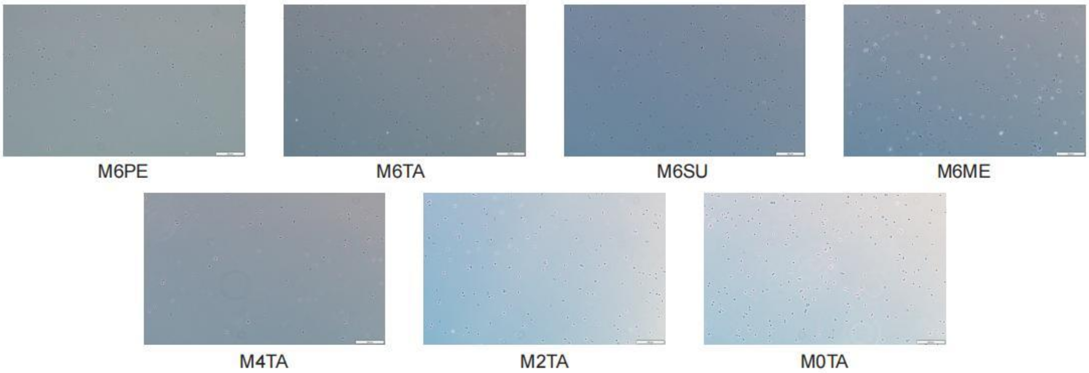

Supplementary Figure 9. Microscopic observation of nuclear quality in different groups. Scale bar represents 200 μm.
